# Sustained type 1 diabetes self‐management: Specifying the behaviours involved and their influences

**DOI:** 10.1111/dme.14430

**Published:** 2020-12-08

**Authors:** K. Hamilton, S. H. Stanton‐Fay, P. M. Chadwick, F. Lorencatto, N. de Zoysa, C. Gianfrancesco, C. Taylor, E. Coates, J. P. Breckenridge, D. Cooke, S. R. Heller, S. Michie, the DAFNEplus study group

**Affiliations:** ^1^ Centre for Behaviour Change University College London London UK; ^2^ Diabetes Centre King’s College Hospital London UK; ^3^ Sheffield Diabetes and Endocrine Centre Sheffield Teaching Hospitals NHSF Trust Sheffield UK; ^4^ School of Health and Related Research University of Sheffield Sheffield UK; ^5^ School of Nursing and Health Sciences University of Dundee Dundee UK; ^6^ School of Health Sciences University of Surrey Guilford UK; ^7^ Department of Oncology and Metabolism University of Sheffield Sheffield UK

## Abstract

**Aims:**

Sustained engagement in type 1 diabetes self‐management behaviours is a critical element in achieving improvements in glycated haemoglobin (HbA1c) and minimising risk of complications. Evaluations of self‐management programmes, such as Dose Adjustment for Normal Eating (DAFNE), typically find that initial improvements are rarely sustained beyond 12 months. This study identified behaviours involved in sustained type 1 diabetes self‐management, their influences and relationships to each other.

**Methods:**

A mixed‐methods study was conducted following the first two steps of the Behaviour Change Wheel framework. First, an expert stakeholder consultation identified behaviours involved in self‐management of type 1 diabetes. Second, three evidence sources (systematic review, healthcare provider‐generated ‘red flags’ and participant‐generated ‘frequently asked questions’) were analysed to identify and synthesise modifiable barriers and enablers to sustained self‐management. These were characterised according to the Capability‐Opportunity‐Motivation‐Behaviour (COM‐B) model.

**Results:**

150 distinct behaviours were identified and organised into three self‐regulatory behavioural cycles, reflecting different temporal and situational aspects of diabetes self‐management: Routine (e.g. checking blood glucose), Reactive (e.g. treating hypoglycaemia) and Reflective (e.g. reviewing blood glucose data to identify patterns). Thirty‐four barriers and five enablers were identified: 10 relating to Capability, 20 to Opportunity and nine to Motivation.

**Conclusions:**

Multiple behaviours within three self‐management cycles are involved in sustained type 1 diabetes self‐management. There are a wide range of barriers and enablers that should be addressed to support self‐management behaviours and improve clinical outcomes. The present study provides an evidence base for refining and developing type 1 diabetes self‐management programmes.

**Novelty statement**

- Type 1 diabetes self‐management programmes improve HbA1c in the short‐term, but participants may struggle to maintain behavioural changes.
- This is the first study to map key diabetes self‐management behavioural processes using the Behaviour Change Wheel.
- Three cycles of self‐management behaviours were developed: Routine, Reactive and Reflective. 34 barriers and 5 enablers of these behaviours were identified, relating to Motivation (e.g. ‘Discomfort managing diabetes in public’), Capability (e.g. ‘Lack of skills to apply principles’) and Opportunity (e.g. ‘Group experiences adding credibility’).
- This work will inform refinement of self‐management programmes to support long‐term behaviour change by incorporating techniques targeting barriers/enablers of maintenance.

## 1 INTRODUCTION

Self‐management is recognised as a central component of diabetes care. It has been estimated that people with diabetes are responsible for 95% of their care.^1^ Self‐management is a broad concept involving cognitive, emotional and behavioural self‐regulatory processes.^2^ Type 1 diabetes is behaviourally demanding and complex, with self‐management involving calculation and administration of insulin doses informed by blood glucose monitoring and review, estimated carbohydrate intake and consideration of other factors such as physical activity levels (e.g. 3, 4, 5). Sustained enactment of self‐management behaviours is recognised as critical to optimising clinical outcomes.^3^, ^4^ While previous work has described behaviours involved in type 1 diabetes self‐management (e.g. 6), relationships between the behaviours and the order in which these need to be carried out to increase likelihood of improvements in blood glucose levels are undefined.

In the UK, National Institute for Health and Care Excellence (NICE) guidance recommends that adults with type 1 diabetes are offered self‐management support in the form of a structured education programme.^5^ One recommended programme is Dose Adjustment for Normal Eating (DAFNE), which has been widely adopted nationally and internationally, and is described in detail elsewhere (see 8, 9). It is a skills training course that advocates a regimen of flexible intensive insulin therapy, promoting freedom in dietary choices. While DAFNE is effective in improving quality of life and reducing HbA1c in the months following participation, improvements in HbA1c are not typically sustained beyond six to 12 months,^6^, ^7^, ^8^ although see.^9^ This suggests there may be scope to promote sustained self‐management by refining DAFNE and other similar programmes, drawing on behavioural science.^10^, ^11^, ^12^

Intervention development frameworks from behavioural science offer a systematic approach to refining interventions. The Behaviour Change Wheel is one such framework, which is based on a synthesis of intervention frameworks and has the COM‐B (Capability‐Opportunity‐Motivation‐Behaviour) model at its hub.^13^ The framework has been applied to refine existing interventions aiming to change health‐related behaviours.^14^, ^15^, ^16^ The first step in the Behaviour Change Wheel process is identifying and precisely specifying the behaviour(s) underpinning the outcome of interest. The second step is identifying the key influences on these behaviours, and what it would take to bring about change.^17^ The COM‐B model^13^ presents an integrated way to systematically explore behavioural influences. It specifies three necessary conditions for a behaviour to occur: capability (i.e. knowledge and skills), opportunity (i.e. physical and social environment), and motivation (i.e. reflective, or conscious, and automatic reactions).

In this study the Behaviour Change Wheel framework was applied to 1) specify the behaviours involved in sustained type 1 diabetes self‐management, and 2) identify the influences on these behaviours. This a pre‐requisite for subsequently identifying behaviour change techniques and intervention components that address identified barriers and enablers and facilitate sustained self‐management behaviours. This study was part of a wider programme of research, DAFNE*plus*, in which behavioural science was used to optimise the DAFNE self‐management programme to improve clinical outcomes and quality of life for people with type 1 diabetes.^18^

## 2 METHODS

### 2.1 Design

**FIGURE 1:**
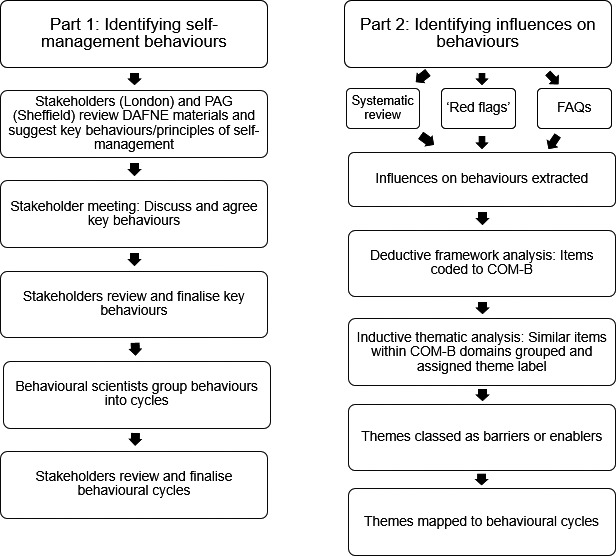
Overview of methodological steps. (NB Part 2 steps were undertaken by two researchers independently, with reliability assessed using member checking)

A two‐part mixed‐methods study was conducted corresponding to the first two stages of the Behaviour Change Wheel process for developing or refining behaviour change interventions.^17^ Part 1 involved expert stakeholder consultation to identify the behaviours involved in self‐management advocated by the DAFNE programme. Part 2 involved identifying barriers and enablers to enacting these self‐management behaviours across multiple evidence sources and categorising these according to COM‐B (see Figure 1 for an overview). Ethical approval for this study was granted by the University College London Division of Psychology and Language Sciences Ethics Committee (CEHP/2013/508).

### 2.2 Identifying type 1 self‐management behaviours according to DAFNE

A face‐to‐face consultation meeting held in London to identify the behaviours involved in the self‐management of type 1 diabetes was attended by 16 stakeholders: five clinical diabetologists with experience of delivering or developing the DAFNE programme, three specialist nurse and dietitian DAFNE educators, six health and clinical psychologists and behavioural scientists with expertise in type 1 diabetes (two of whom had type 1 diabetes) and two independent representatives of the type 1 diabetes patient advisory group who had previously attended a DAFNE course. Prior to the stakeholder meeting, a patient advisory group meeting attended by five people with diabetes and two clinicians was held in Sheffield to discuss the DAFNE programme and the ‘fundamental principles of self‐management’ (defined as things people had to do or know in order to be able to self‐manage effectively). These principles were added to the actions generated by the stakeholder meeting. Stakeholders were asked to review the most recent DAFNE course curriculum prior to the meeting and to suggest the actions (i.e. behaviours) involved in self‐management of type 1 diabetes, based on their review of the curriculum and their diabetes experience. After the face‐to‐face meeting, stakeholders were sent this list for review and asked to add any actions that might have been missed.

Four behavioural scientists subsequently met to group the behaviours generated into themed topics. For example, ‘calculating carbohydrate content by weighing’ and ‘Using resources to identify carbohydrate content of foods’ were put into the group ‘Carbohydrate‐Counting’. In recognition of the interdependent nature of the behaviours involved in self‐management, and high level of self‐regulation required to maintain them (i.e. monitoring behaviour/outcomes in relation to a goal and acting to correct discrepancies), these topics were then organised into flow diagrams representing cycles of behaviours. Stakeholders were invited to comment on the diagrams via email or in face‐to‐face meetings, and suggested refinements to ensure the cycles represented the self‐regulatory processes involved in diabetes self‐management as accurately as possible. The final self‐management behaviours and diagrams were circulated to all stakeholders for approval.

### 2.3 Identifying influences on self‐management behaviours

Influences on the target self‐management behaviours were extracted by two researchers (KH and SSF) from three sources. The sources were:

#### 2.3.1 Systematic review of published literature

We extracted the reported barriers and enablers to self‐management following participation in DAFNE from the findings of a systematic review and meta‐ethnography,^19^ which reviewed 18 articles reporting on six qualitative studies of people with type 1 diabetes who had taken part in a DAFNE course.

#### 2.3.2 Red flags

Seven DAFNE educators from three NHS Trusts (with on average 16 years’ experience delivering DAFNE courses) were asked to list factors (characteristics, situations, course experiences etc.) based on their clinical experience that represent ‘red flags’ that might indicate that a participant may need additional support with self‐management at three time points: before, during and after participation in a DAFNE course.

#### 2.3.3 FAQs

The DAFNE educators were also asked to create a list of frequently asked questions (FAQs) that participants typically ask after attending a DAFNE course, drawing on their clinical experience and by asking participants via the DAFNE Online forum to report aspects of self‐management they were unsure of or struggled with post‐course.

#### 2.3.4 Data analysis

Extracted barriers and enablers from the systematic review, red flags and FAQs were categorised according to the COM‐B model using a combined deductive framework analysis and inductive thematic analysis approach (following the methods of Graham‐Rowe et al. ^20^.

*Deductive framework analysis*: Extracted data were deductively coded to the COM‐B domain(s) they were judged to represent. For example, ‘Repeatedly failing to achieve blood glucose targets is demoralising and results in feelings of failure’ was coded under the domain ‘automatic motivation’ (i.e. emotions). Coding was conducted in MS Excel by two researchers independently (KH/SSF) and any disagreement or uncertainty resolved via discussion with a third researcher (FL).

*Inductive thematic analysis*: Each COM‐B domain was considered in turn. Similar items coded to the same domain were grouped together and a summary theme label was inductively generated, with any disagreements resolved as above. For example, the items ‘Do I need to retest after a hypo?’ and ‘If my BG is below 3.5 at my mealtime, should I eat first then inject?’ (both FAQs) were both coded to the domain Psychological Capability, grouped together and summarised using the inductively generated theme label ‘*Lack of skills to apply DAFNE principles’* within the subtheme ‘*Lack of knowledge*/*skill about how to treat a hypo’*. Resulting themes were classified as either a barrier or an enabler by looking at the qualitative meaning of the data points contributing to that theme, and whether the data suggested that this factor hindered or sustained self‐management. Themes were then mapped to the corresponding behaviour(s) identified in Step 1. For example, the theme ‘*Perception of hypoglycaemia as an opportunity to overindulge in unhealthy foods*’ was classified as a barrier and mapped to the behaviour ‘Carry out and record hypoglycaemia treatment’ in the Reactive cycle (see Figure 1 for an overview of methodological steps).

## 3 RESULTS

### 3.1 Identifying self‐management behaviours

**FIGURE 2:**
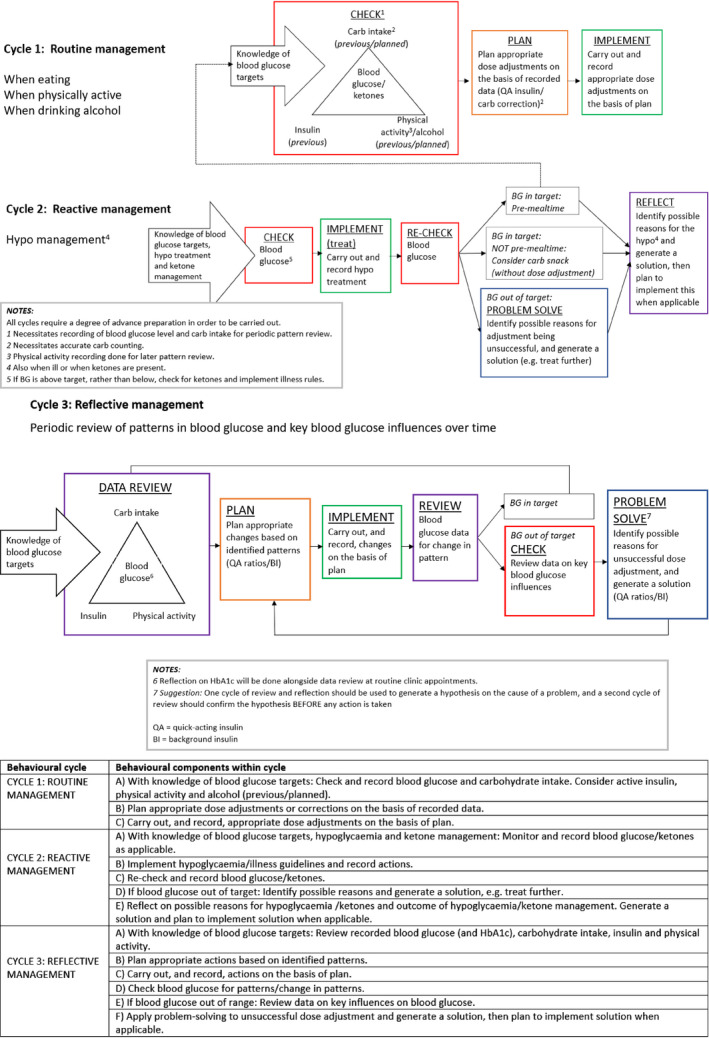
Type 1 diabetes self‐management behavioural cycles

Stakeholders identified 150 behaviours needed for optimal and sustained self‐management of type 1 diabetes. These were combined into three behavioural cycles reflecting the different temporal and situational aspects of diabetes self‐management (see Figure 2).

#### 3.1.1 Cycle one: Routine self‐management

This cycle describes the adaptive self‐management behaviours required to keep blood glucose within target on a daily basis, influenced by food intake, physical activity and alcohol consumption. The steps involve daily checking, planning and implementing a response, based on regular data gathered. This cycle may need to be enacted several times a day in response to fluctuating parameters.

#### 3.1.2 Cycle two: Reactive self‐management

This cycle describes the self‐management behaviours required to manage the impact of unanticipated events that have an impact on blood glucose regulation, e.g. hypoglycaemia or suspected illness or presence of ketones. The steps involve checking, re‐checking, problem solving, reflecting and planning. This cycle is in response to an atypical event and incorporates actions to reduce the incidence of these events where possible.

#### 3.1.3 Cycle three: Reflective self‐management

This cycle describes the longer‐term review of blood glucose data (ideally on a weekly basis) to evaluate the impact of daily self‐management behaviours. The steps involve planning, implementing, reviewing, monitoring and problem solving. This cycle supports the fine‐tuning of habitual self‐management behaviours by identifying patterns in blood glucose data, and the instigation of more long‐term adjustments such as changing background insulin dose or mealtime ratios.

### 3.2 Identifying influences on self‐management behaviours: Barriers and enablers

The stakeholder consultation produced 97 FAQs and 69 red flags; 45 barriers and enablers were extracted from the literature review. This resulted in 211 data points overall. These were synthesised into 39 themes representing barriers and enablers to self‐management behaviours, coded to COM‐B. Thirty‐four of these represented barriers to sustained self‐management and five represented enablers. These are presented in Table 1 according to each domain of COM‐B and summarised below. All barriers and enablers mapped to target behaviours can be found in Appendix 1.

**TABLE 1:** Influences on self‐management behaviours: Themes of barriers and enablers to DAFNE self‐management behaviours, categorised according to the COM‐B model

COM‐B domain	Theme	Direction of influence	Behavioural cycle component influenced^a^	Source	Example data point
Capability					
*Physical*	Impaired hypoglycaemia awareness/symptoms	Barrier	2A	SR, RFs, FAQs	Why aren't my warnings as good as they used to be?
	Physical/cognitive hypoglycaemia symptoms causing over‐treatment	Barrier	2B	SR	The panic, disorientation, lack of concentration and increased hunger during an episode can lead to over‐treatment.
	Physical symptoms inhibiting monitoring	Barrier	1A, 2A, 2C	FAQs	My fingers are getting really sore, can I stop testing to let them recover?
*Psychological*	Establishing and maintaining routines	Enabler	All	SR, FAQs	Some participants impose a routine such as setting an alarm to administer their BI at the same time of day, even on weekends.
	Difficulty incorporating DAFNE principles into everyday life and challenges	Barrier	All	FAQs, RFs, SR	Participants who do not lead routinised lives with predictable working patterns and regular mealtimes struggle to integrate self‐management practices into their everyday lives.
	Lack of longer‐term pattern review strategy due to reliance on corrective doses	Barrier	3A, 3B, 3C, 3D, 3E, 3F	SR, RFs, FAQs	Many participants come to rely predominantly on simpler corrective doses to achieve target glucose levels rather than reviewing glucose profiles and using these to alter their background insulin doses or mealtime ratios.
	Forgetting exact blood glucose targets	Barrier	1A, 2A, 2C, 3A, 3D	SR	Many participants shift their blood glucose targets upwards over time, whether consciously or inadvertently because they struggle to remember them, resulting in their re‐instating those used pre‐course.
	Forgetting injection sites	Barrier	1C	RFs	Forgetting injection sites.
	Poor numeracy/ literacy	Barrier	All	RFs, SR	Unable to grasp principles due to literacy/numeracy.
	Lack of skills to apply DAFNE principles	Barrier	All	FAQs, RFs, SR	Many participants do not have the skills needed to change the settings independently in the event that their mealtime insulin requirements change.
					What should I do if my BGs are high on waking?
					If my BG is below 3.5 at my mealtime, should I eat first then inject?
Motivation					
*Automatic*	Anxieties/ fears	Barrier	All	SR, RFs	Anxiety from past diabetes experiences
	Feelings of failure and hopelessness	Barrier	All	SR	Repeatedly failing to achieve BG targets is demoralising and results in feelings of failure.
	Perceived burden of self‐management behaviours	Barrier	All	FAQs, SR	DAFNE makes me think about my diabetes all the time. Is it always going to be like this?
	Lack of acceptance of diagnosis	Barrier	All	RFs	Those who cannot accept their diagnosis
	Discomfort managing diabetes in public	Barrier	1A, 1C, 2A, 2C, 3A, 3C, 3D, 3F	FAQs	How can I feel less embarrassed about my hypos?
	Wanting timely and tangible rewards for self‐management efforts	Barrier	All / 1A	SR	Participants want tangible rewards for self‐management efforts, and when there is disconnect between effort and reward they become demotivated and frustrated.
*Reflective*	Feeling empowered by new knowledge and skills	Enabler	All	SR	New knowledge (e.g. better understanding of condition, rules to follow) and skills means participants feel more confident and better equipped to manage diabetes.
	Lack of confidence applying skills/DAFNE principles independently	Barrier	All	SR, RFs	Participants question their ability to review blood glucose readings, interpret patterns and make adjustments to background insulin doses and mealtime ratios.
	Lack of belief about need for monitoring	Barrier	1A, 2A, 2B, 2C	FAQs	I can tell if my BG is too high or low, so why do I need to test?
	Lack of belief in health consequences of poorly controlled diabetes	Barrier	All	FAQs	My HbA1c is good, but my BGs are erratic, does it matter?
	Uncertainty about the implications of following DAFNE principles	Barrier	All	FAQs	Will I put on weight once I have got my BG levels down?
	Perception of hypoglycaemia as an opportunity to overindulge in unhealthy foods	Barrier	2B	SR	A few participants report using hypoglycaemia as an excuse to overindulge in foods that they enjoy.
	Reluctance to over‐burden HCPs	Barrier	All	SR, RFs	Someone who doesn't like to bother HCPs because they think they are too busy
	Perception that blood glucose targets are not achievable	Barrier	1A, 2A, 2C, 3A, 3D	SR, FAQs	Are these targets realistic? How many people in DAFNE achieve them?
	Lack of trust in guidelines	Barrier	2B	SR	Some participants purposefully over‐treat hypoglycaemia because they do not trust the treatment amounts specified on the course.
	Having clear targets and guidelines	Enabler	1A, 1B, 2A, 2B, 2D, 3A, 3E	SR	Patients find use of blood glucose targets motivational. Targets enable patients to identify problems with blood glucose control and prompt them to make insulin dose adjustments independently, or with assistance.
	Lack of diabetes prioritisation in the face of life events and challenges	Barrier	All	SR, RFs	At critical junctures (e.g. illness and bereavement) participants can intentionally or unintentionally prioritise other areas of life resulting in less rigid application of self‐management practices.
	Lack of intention to follow DAFNE principles	Barrier	1A, 2A, 2C, 3A	RFs, SR	People who don't fill in a diary
	Lack of readiness to change	Barrier	All	RFs	Reluctance to try new theories/practices
	Didactic culture of healthcare inhibiting independent decision‐making	Barrier	All	SR	Many participants still prefer to defer self‐management decisions to health professionals.
Opportunity					
*Physical*	Lack of access to appropriate support	Barrier	All	SR, RFs	Participants find it confusing and disheartening when health professionals unfamiliar with the DAFNE approach ‘over‐rule’ their newly acquired expertise, resulting in some avoiding contact with mainstream services altogether.
	Technology/ bolus adviser assisting application of DAFNE principles	Enabler	1A, 1B, 1C, 2A, 2B, 2C	SR	Using an automated bolus advisor can reduce the burden of data recording, and apps that link the bolus advisor to participants’ smartphones may be an even more convenient means of record keeping.
	Bolus adviser eroding manual adjustment skill upkeep	Barrier	1A, 1B, 1C, 2A, 2B, 2C	SR	Over‐reliance on a bolus advisor can prevent participants from developing their mathematical skills and taking greater control over self‐management.
	Inadequate access to monitoring equipment	Barrier	2A, 2B	FAQs	I've run out of ketone strips, does it matter?
	Chaotic, unstable or non‐routine lifestyle	Barrier	All	SR, RFs, FAQs	No fixed abode ‐ does not know what and when next meal is going to be. No cooking facilities, no weighing scales, etc.
*Social*	Inappropriate social support	Barrier	All	SR	Many participants seek support from significant others, but friends and family often lack knowledge about diabetes management, leaving participants feeling confused as they attempt to implement new practices.
	Perceived social stigma of managing diabetes (in public)	Barrier	1A, 1C, 2A, 2C, 3A, 3C, 3D, 3F	FAQs	I don't like testing my blood glucose/injecting in front of other people. What do I do when I go out to eat?
	Negative comparison with others’ progress	Barrier	All	RFs	Seeing positive changes in others’ BG control whilst the person in question has worsening control (hypos/hypers) resulting in a lack of confidence in themselves and DAFNE.
	Group experiences adding credibility	Enabler	All	SR	The accumulation of experiences which patients bring to the group helps to add credence and credibility to key teaching points on the curriculum.

Abbreviations: BG = blood glucose; FAQs = frequently asked questions; RFs = red flags; SR = systematic review.

^a^ 1 = Routine cycle; 2 = Reactive cycle; 3 = Reflective cycle. See Figure 1 for cycle components.

#### 3.2.1 Physical capability

All three themes within this category were barriers, and related to physical symptoms. For example, ‘impaired awareness of hypos’ is a barrier as the lack of physiological cues does not provide a trigger for checking blood glucose and treating of hypoglycaemia.

#### 3.2.2 Psychological capability

Six themes within this category were barriers, and included difficulty adapting self‐management routines in the face of changing life events such as new caring responsibilities. There was only one enabler: establishing and maintaining routines, which diminishes the cognitive burden of behavioural change and reduces the likelihood of behavioural sequences being disrupted. Many of these themes influenced all self‐management behaviours as they related to sub‐optimal DAFNE knowledge and skills, for example assessing and adjusting meal‐time ratios or managing physical activity. (See the Appendix for a full list of behaviours).

#### 3.2.3 Automatic motivation

All six themes within this category were barriers, for example anxiety about managing diabetes and acute symptoms, or a lack of acceptance, both leading to disengagement with self‐management behaviours in order to reduce emotional discomfort associated with diabetes. Again the majority of these barriers related to all self‐management behaviours, as they could contribute to a global withdrawal from self‐management.

#### 3.2.4 Reflective motivation

Of the 14 themes in this category, 12 were barriers, such as a lack of trust in guidelines, leading to people modifying their goals (e.g. blood glucose targets). Two were enablers, relating to feeling empowered by new knowledge and skills, which leads to confidence in independent decision‐making, and having clear guidelines and targets to aim for. The majority of themes related to all behaviours, such as a lack of prioritisation of diabetes self‐management when faced with competing demands, or a lack of readiness to change, causing disinclination to enact certain principles.

#### 3.2.5 Physical opportunity

This category contained four barriers, such as inadequate access to monitoring equipment (e.g. blood glucose meter strips), which impairs ability to follow blood glucose monitoring guidelines. One enabler was the presence of bolus advisers facilitating data recording, which increases the likelihood of timely recording by reducing the associated cognitive burden. Several themes, such as lack of a stable home environment, related to all self‐management behaviours.

#### 3.2.6 Social opportunity

Three themes in this category were barriers relating to social influences beyond the DAFNE setting, such as the perception of external criticism, influencing behaviours such as injecting insulin in public before eating. The single enabler related to the DAFNE group learning environment, with participants’ shared experiences providing validation of and credibility to information given by educators. Most themes influenced all self‐management behaviours.

## 4 DISCUSSION

This study identified 150 distinct behaviours related to sustained self‐management in type 1 diabetes, summarised into three interdependent cycles of behaviour (Routine, Reactive, Reflective), and 39 barriers and enablers to these behaviours. Most of the behaviours within each cycle have been identified in previous research,^21^ providing verification of these behaviours. This study also systematically breaks down the sequence in which behaviours need to be carried out in order to faciltiate participant learning and retention. Most barriers and enablers identified related to Reflective Motivation, followed by Psychological Capability and Automatic Motivation, reinforcing the links established in previous studies between psychosocial factors, self‐management and clinical outcomes.^22^

The findings from this study highlight a number of potential ways to optimise type 1 diabetes self‐management programmes. First, conceptualising self‐management behaviours in terms of functionally connected cycles recognises the complexity of type 1 diabetes self‐management in the moment (within the Routine and Reactive cycles) as well as acknowledging the importance of reflecting on self‐management over a longer period (within the Reflective cycle). The high number of distinct behaviours identified, reflects the notion that sustained self‐management in type 1 diabetes is a complex set of behaviours, consisting of multiple sub‐behaviours. People may struggle with certain behaviours more than others. Evidence reported from other studies of people with diabetes^23^ as well as representatives and clinicians in our stakeholder group suggests that the Reflective cycle is the most challenging for people. This points to a need for a more targeted and tailored approach to designing self‐management programmes in type 1 diabetes. This should begin with identifying which specific behaviours the individual is finding challenging, setting behaviourally focused goals (rather than goals around improving self‐management more broadly), and selecting strategies to address the barriers and enablers uniquely associated with selected behaviours. Although current DAFNE teaches pattern recognition, this study highlights the need to promote the *benefit* of reflective over spontaneous decision‐making and furthermore, to provide skills in impulse control and emotion regulation that would enable this process to occur in real life. The behavioural cycles developed as part of this study could also be used in a clinical context during consultations with people with diabetes to facilitate consideration and identification of target behaviours and to provide a temporal order of skill acquisition, starting with routine daily adjustments and moving onto less frequent and more specific blood glucose management scenarios.

Second, the large number of themes within the COM‐B domain of Motivation suggests that self‐management of type 1 diabetes longer‐term may be driven predominantly by emotional and cognitive influences. In particular, anxiety in relation to diabetes was identified as a key influence in automatic motivation (i.e. anxiety related hypoglycaemia, general health, ‘getting it wrong’). While fear of hypoglycaemia has received much research attention, the impact of anxiety about complications has been less studied, despite research suggesting it may be at least as significant a concern.^24^ It is possible that fear of complications could prompt withdrawal from diabetes self‐management through reducing acceptance, or conversely that it could act as a motivating factor to keep blood glucose in range. This highlights the need for self‐management interventions to extend beyond educational and training strategies focused on short‐term knowledge and skill acquisition, towards inclusion of psychological support to help manage anxiety, bolster resilience, and enhance and maintain motivation longer term (e.g. habit formation, persuasion, enablement). This will involve addressing the identified cognitive and emotional experiences (beliefs, perceptions, anxieties, concerns, and acceptance) associated with living with the condition and affecting engagement in self‐management behaviours.

Ensuring that the philosophy underpinning self‐management programmes, such as DAFNE, are person‐centred, flexible and tailored to the needs, values and motivations of the individual participants is critical to ensuring successful delivery and continued engagement.^25^ Multi‐disciplinary working and adhering to principles of care planning support this approach. Ongoing training and support for health care professionals in these approaches is also required and an area for future research.

Use of the Behaviour Change Wheel framework^17^ provides a method for systematically selecting strategies to address the key identified barriers and enablers to self‐management. The COM‐B model is mapped to the Behaviour Change Wheel which specifies nine broad ways to change behaviour (i.e. persuasion, enablement, environmental restructuring), as well as a taxonomy of 93 more granular behaviour change techniques (BCTs), in order to suggest the types of interventions likely to be effective in addressing different types of barriers and enablers^17^ Potential BCTs that could be included in future or revised interventions to support sustained self‐management in type 1 diabetes are summarised in Table 2.

**TABLE 2:** Example Behaviour Change Wheel process to identify behaviour change techniques and intervention components to address identified barriers and enablers to self‐management

Theme	COM‐B domain	Example intervention function	Example behaviour change technique	Example intervention component
Physical symptoms inhibiting monitoring	Physical capability	Training	Behavioural practice/rehearsal	Practise rotating sites for checking blood glucose, to avoid sore spots
Forgetting exact blood glucose targets	Psychological capability	Environmental restructuring	Prompts/cues	Provide cue cards of blood glucose target ranges to be kept where they may be most helpful, e.g. with bolus adviser/insulin
Feelings of failure and hopelessness	Automatic motivation	Persuasion	Focus on past success	Highlight and draw on any success in previous behaviours, e.g. monitoring
Lack of intention to record data	Reflective motivation	Persuasion	Information about health consequences	Provide information about the health consequences of having accurate data to aid in decisions
Technology/bolus adviser assisting application of DAFNE principles	Physical opportunity	Enablement	Restructuring the physical environment	Provide a bolus adviser and appropriate software to aid in calculating and recording insulin doses and carbohydrate intake
Inappropriate social support	Social opportunity	Enablement	Social support (unspecified)	Provide information on and examples of how to identify unhelpful social support, and how to elicit and helpful support

Such strategies will be evaluated in the DAFNE*plus* research programme,^18^ which aims to refine the existing DAFNE self‐management programme to include BCTs targeting the behaviours and influences to sustained self‐management identified in this study (Stanton‐Fay et al., under review).

This work could also help strengthen self‐management research by feeding into the refinement of diabetes specific measures such as the Summary of Diabetes Self‐Care Activities measure^23^ or the Diabetes Self‐Management Questionnaire.^4^ As yet, there is no tool available for assessing engagement in self‐management behaviours specific to type 1 diabetes. However, work to develop such a questionnaire is currently underway: Self‐Care Behaviours in Type 1 Diabetes (SCB‐T1D, Cooke et al., in prep.).

In addition to the grounding in behavioural science theories and frameworks, a key strength of this study is the use of triangulation, incorporating perspectives from clinicians, people with diabetes and researchers. People living with diabetes and clinicians are able to offer different insights into challenges and factors enabling self‐management; a review of relevant studies concluded that co‐production (involving users in the evaluation or design of services) is particularly valuable when generating ideas for healthcare change.^26^ One limitation, is that this study involved a secondary analysis of the published findings of a previous review on barriers and facilitators for sustaining self‐management skills after attending a type 1 diabetes structured education programme.^19^ This introduces a source of potential bias, in that the original data had already undergone a degree interpretation and synthesis by the review authors. However, involvement of the senior author of that review (JB) in the current study's analysis allowed opportunity for discussion and clarification to ensure similarity in data interpretation. Furthermore, none of the studies included in the review explored barriers and enablers to self‐management using a theory of behaviour change. It is therefore possible that they did not explore the full range of potential influences on self‐management behaviours, highlighting the need for more theory‐based studies in this field.

In conclusion, this study identified three cycles of self‐management behaviours: Routine, Reactive and Reflective, translating the complexity and self‐regulatory nature of self‐management into a useable framework. We found enactment of these cycles to be influenced by 39 themes relating to Capability, Opportunity and Motivation; 35 of which acted as barriers to self‐management and five of which acted to enable self‐management. This work is the first to the authors’ knowledge to articulate the influences on self‐management of type 1 diabetes using the Behaviour Change Wheel framework. It provides a model for considering the behavioural processes underpinning long‐term conditions that require enactment of self‐management behaviour, with a view to developing targeted interventions, including that used in the DAFNE*plus* intervention development programme.^18^

## CONFLICT OF INTEREST

SRH has undertaken consultancy for Eli Lilly, Novo Nordisk, Sanofi Aventis, Zealand Pharma, manufacturers of analogue insulins and treatment for hypoglycaemia for which his institution has received remuneration. He has also served on speaker panels for Novo Nordisk for which he has received remuneration. The remaining authors have no conflict of interest to declare.

## Supporting information

Supplementary Material
